# With whom do you feel most intimate?: Exploring the quality of Facebook friendships in relation to similarities and interaction behaviors

**DOI:** 10.1371/journal.pone.0176319

**Published:** 2017-04-28

**Authors:** Jieun Wee, Joonhwan Lee

**Affiliations:** Department of Communication, Seoul National University, Seoul, Republic of Korea; University of Rijeka, CROATIA

## Abstract

It is widely accepted that people tend to associate more and feel closer to those who share similar attributes with themselves. Most of the research on the phenomenon has been carried out in face-to-face contexts. However, it is necessary to study the phenomenon in computer-mediated contexts as well. Exploring Facebook is important in that friendships within the network indicate a broader spectrum of friends, ranging from complete strangers to confiding relations. Also, since diverse communication methods are available on Facebook, which method a user adopts to interact with a “friend” could influence the quality of the relationship, i.e. intimacy. Thus, current research aims to test whether people in computer-mediated contexts do perceive more intimacy toward friends who share similar traits, and further, aims to examine which interaction methods influence the closeness of relationship by collecting activity data of users on Facebook. Results from current study show traits related to intimacy in the online context of Facebook. Moreover, in addition to the interaction type itself, direction of the interaction influenced how intimate users feel towards their friends. Overall findings suggest that further investigation on the dynamics of online communication methods used in developing and maintaining relationships is necessary.

## Introduction

People form and maintain relationships with one another. Yet, not all relationships are identical; they differ in intimacy, which defines the quality of the relationships. Among many possible explanations on why people show different communication behaviors in different relationships, an extensive literature established by social scientists manifests an approach based on the tendency of homophily, i.e., “birds of a feather flock together.” According to the literature, people tend to feel more attached to others with similar attributes, associate more often with similar people, and as a result, develop closer relationships with them [1]. The majority of research on elaborating the phenomenon has been conducted within the offline context [2–5], and a small portion has been dedicated to the online context [6, 7].

However, more attention should be given to understanding online communication behaviors. People exchange information, debate controversial issues, and have casual conversations online as they do face-to-face. In doing so, Social Network Services(SNSs) such as Twitter and Facebook facilitate interactions not only by providing spaces to initiate and develop relationships, but by providing multiple ways to actually affiliate with others. In nature, relationships observed in SNSs are distinct from those observed in face-to-face in that online relationships are often formed between people who have never met each other in person before. Moreover, relationships may differ even within the online context depending on the provided platform or features available within the service. For example, while relationships built in Twitter as “following” and being followed do not require technical reciprocation and often are not directly connected to offline relationship, relationships on Facebook require mutual confirmation on relationships and are often based on offline relationships.

Though personal networks on Facebook are mostly based on already-existing relationships [8, 9], Facebook friends are different from *friends* that we ordinarily refer to in our everyday lives. Facebook friends indicate people connected through the service including complete strangers, latent ties, and close friends; yet, traditional meaning of a friend implicates something more than a mere connection, i.e. mutual affection. In other words, it could be understood that relationships on Facebook marked as friends cover a broader spectrum than friends within offline contexts. People form relationships based on different levels of intimacy or sometimes even without any intimacy, but are all still called equally as friends on Facebook. Elaboration on the relationships in Facebook is particularly important because the observation may provide insights on the similarities and differences in communication behaviors between online and offline contexts. Not only does Facebook reflect users’ offline relationships within the computer-mediated context, but also show distinct online communication behaviors such as “messaging,” “liking,” and “sharing.” Moreover, Facebook stores enormous information on such behaviors, enabling observation on interaction that occur in the process of relationship formation and maintenance. Thus careful examination on communication behaviors and relationships in Facebook is crucial.

By looking into the similarities and interaction behaviors on Facebook, current research explores whether sharing similar attributes relate to higher intimacy in online relationships (Research Question 1). Further, it investigates how each of different communication methods available in the online space contributes to developing intimacy (Research Question 2). From a perspective of empirically looking into relationships as a dynamic process rather than a static state, keeping relations could be observed in terms of seeking continued contact with the counterpart, i.e. interacting with each other [1]. Beyond similarities between users, since people affiliate online via various communication methods, we assume that certain interaction behaviors would be prominent in enhancing intimacy online. The second research question is particularly meaningful in that it correlates self-reported survey data on intimacy and actual behavioral data on interaction as an attempt to provide a complementary explanation on computer-mediated relationships, where people communicate with others through numerous manners, e.g. sending chat messages, tagging others in photos, commenting on others’ photos etc.

The term homophily was conceptualized as a tendency in order to encourage the idea that different degrees of affiliation among different kinds of relationships exist, and that the simple expression of “flocking together” lacks clarity in the terminology; demonstrating that the positive correlation between the attribute similarity of people and the quality of relationship could be measured [1]. Research such as similarity-attraction hypothesis claiming that people are generally attracted to those similar to themselves [10], self-categorization theory contesting that people appraise others as similar and dissimilar to themselves by categorizing their own attributes and comparing such with others’ attributes [11], and studies probing similarity as a variable on the social tie formation are all in the same context [2, 12]. Accordingly, the concept presumes that the effect of the affiliating tendency would be greater when people share multiple attributes concurrently than when they share a single attribute.

The earliest studies on the inclination of people to associate more with those who share personal traits were focused on small social groups of college students, adolescents from school classes, and urban neighborhoods through ethnographic observations in the early 1900s [5]. Throughout the mid 1900s, though subjects of research expanded to other public places, it still had restrictions on the observable range since the studies depended solely on observation of researchers. Modern sample surveys were applied to the studies in the 1970s. This methodology not only made empirical research on relationship formation and maintenance among large-scale groups such as schools and communities feasible, but also made simultaneous measure on multiple characteristics of individuals possible by self-reporting [13, 14]. Since the late 1980s, many studies attempted to provide more concrete empirical evidence on the phenomenon through longitudinal research. Hallinan and Smith studied how inter-racial friendships among classmates change by self-reported friendship nomination of participants [15]. Further, Burt conducted research on how relationships among colleagues decay throughout time by implementing surveys [16].

Among diverse attributes that compose a person, those considered in determining similarities that influence the nature of relationships are often limited to the following: race/ethnicity, sex/gender, age, education, occupation, and social class. In a longitudinal study of cross-race and inter-race friendships in elementary classrooms, gender was reported as a more powerful predictor than race (black or white) in case of close friendships, and that gender even strengthens cross-race friendship choices over time [15]. Considering contradictory findings from other research that shows stronger effects of race and ethnicity than sex and gender unless in sex-segregated structure [17], the result from Hallinan and Smith’s study may be due to the participants’ age—children from an elementary school [15]. Generally, race and ethnicity are the most salient dimensions that indicate psychological closeness across a wide range of relationships, from the most intimate relationships of marriage to merely acquaintances [3, 5, 14]. Though not as strong as race and ethnicity, religion has a significant effect for those who have intimate relationships, when not involving kin [3, 13]. Being in a similar age tends to strengthen intimacy, though the effect weakens as people get older [5, 14]. Highly due to the strong influence of propinquity set by the surroundings, education, occupation, and social class generate greater affiliation; education on school setting, occupation on workplace, and social class on residential area [5]. Yet the effects are considered to be less significant than race and ethnicity [3]. Some studies show that education, occupation, and social class tend to have greater effects on less intimate relationships [2, 5].

However, by investigating single attributes separately such as the effect of age and effect of gender respectively, previous studies have not measured the effect of relative attribute similarity considering multiple attributes altogether. Such measure is necessary because it is not a single attribute that influences relationships, but multiple characteristics varying in the similarity. Moreover, depending on specific situations in which people are put, particular attributes become prominent among multiple layers of their social identities—defined as group membership in categories such as age, sex, religion, and ethnicity [18]. Hence, adding up the number of common attributes in order to measure similarities between people disregards contexts in which people are situated. Particularly, in computer-mediated settings, individual traits that used to be obvious and personally verifiable has become less prominent, more deceivable, and even invisible. Even within the online space, the effect of attributes may differ among communication platforms-such as blogs, SNSs, online shopping sites-since each service or website posses distinctive characteristics.

Therefore, it is essential to probe how people form and maintain relationships within online communication platforms. The first research question in current paper is established in order to explore which personal attributes influence the quality of relationships and how prominent they are, in an online space of Facebook.

**Research Question 1.** How do similarities on personal attributes—listed in Facebook user profile—correlate to the intimacy level of the user?

Sharing similarities may not be the key in enhancing the quality of a relationship because personal attributes are rather hidden or less prominent in computer-mediated contexts. Alternatively, interaction behaviors may play a central role in developing intimacy between people. In online spaces, it is behaviors that become apparent, not attributes. In other words, what people can truly “see” is how others talk to me via which communication method, rather than who the others are. Compared to similarities, communication activities between people become more visible and are directly revealed to the other in the relationship. Further, because such conversation threads in online space last and compile, interaction record itself becomes the history of a relationship. Indeed, interaction behaviors are important in that without social contact or interaction, relationships cannot be formed or maintained at the first place no matter how intimate or superficial [1].

Accordingly, recent works studied the tendency of homophily by collecting and analyzing computer log data, directly investigating behaviors other than perceptions [7, 19]; communication behaviors such as how often people associate with whom in which ways, other than asking them to report how intimate or close they feel toward the others. Observing behavioral data in explaining how people make sense of their relationship enabled researchers to minimize biases that may occur from ethnographic observations or self-reporting survey methods. Thelwall conducted an empirical research by collecting user data from MySpace in order to look into the impact of personal attributes such as ethnicity, religion, age, sex, marital status, and sexual orientation [7]. Results demonstrate the prosperity of traditional sources of homophily in the online world; overall positive effect on intimacy except for sex. In the study, the tendency to affiliate with others who share certain traits through direct interaction behaviors between them, i.e. commenting on others’ profile pages, was observed [7]. Also, Kossinets and Watts measured the phenomenon by interaction, i.e. e-mail exchanges, in order to identify whether the association of people originated from the similarity effect of individuals’ preference or from the structural foci surrounding individuals [19]. Within an online context of exchanging emails, they aggregated the total similarity by providing equal weight on each attribute and simply adding them up; counting the number of shared attributes [19]. While their work made an attempt to consider multiple characteristics of individuals in measuring similarity, the context in which individuals are situated is yet disregarded.

By investigating whether a positive correlation between similarity and interaction behavior exists, both of the research [7, 19] examined the tendency of similar people to affiliate more. Yet, according to established literature, the quality of relationship is pivotal when discussing the phenomenon [1, 5]. The quality is defined as perceived affection and feelings, and hence, is a variable that is difficult to measure simply by counting numbers or observing methods of interaction (behavioral data). Though perceived intimacy may be related to interaction patterns in some way, those two variables are conceptually distinct in that the former is perception while the latter is behavior. Even when a logical relation between the two are discovered, because various communication methods are available online, intimacy may have different degree of correlations to each of interaction behaviors. Thus, without exploring correlations between intimacy and interaction behaviors in advance, presuming that communication behaviors directly reflect intimacy can be misleading in measuring the quality of relationships between individuals.

Furthermore, while the homophily tendency is often adapted when providing explanations on relationships in previous studies, primarily in offline contexts, the tendency may not be relevant in online contexts. Individual traits—such as race, sex, and age—that used to be obvious and personally verifiable in face-to face settings become less prominent, more deceivable, and even invisible in computer-mediated settings. At the same time, communication strategies that each person can employ became more diversified, explicit, and traceable in online settings. Every action taken online is recorded as log data and a large portion of the data is accessible or trackable by others, considering that online communication highly depends on *writing*. People are explicitly exposed to others’ interaction behaviors. Thus, it is more likely for an individual to recognize the on-going interaction with others than to notice similarities relying on the already-uploaded personal profiles of others.

The difference in intimacy may also exist depending on the communication method itself, i.e. how the other makes contact: for example, whether the other sends a birthday message to an individual by writing a public post or by sending a private message, or by uploading a photo taken together may affect how she perceives the quality of relationship. Nevertheless, earlier studies on Facebook measured the usage in a more general sense by asking questionnaires on surveys such as “In the past week, on average, approximately how many minutes per day have you spent on Facebook [20]?”. In so doing, they may have overlooked diverse communication channels available in Facebook. However, because some types of interactions are considered more important than others, how often users make contact to others (frequency) in which way (type) may differ according to the quality of the relationship [21]. Yet, arbitrarily deciding what interaction types to include and to exclude without knowing what interactions are considered important to users and what are not, a study may lead to findings far from reality. In the case, conducting a data-driven research can provide insights and unexpected patterns that could not have been obtained by reviewing accumulated findings of previous works. Thus, observing the online space of Facebook based on the stored data can possibly yield deeper understandings in the way people affiliate in computer-mediated contexts.

In a recent work of examining strength of relationships in SNSs, researchers underlined that the type of interaction and the likelihood vary according to the strength, and thus, different weight should be given on each interaction type in terms of the psychological attachment [22]. It could be assumed that people will choose different interaction types to communicate with others depending on the level of intimacy towards the partner, because people tend to allocate resources such as time to form or maintain relationships that they regard more important as the resources are limited [22, 23]. Moreover, the way people interact with others and the activities they perform on Facebook depends on their motivation to use Facebook [24]. For example, in a study of Burke, Marlow, and Lento (2010), people in closer relationships directly interacted with their friends by using comments or chat messages, while people simply viewed the “news feed” to check on others in less intimate relationships [24]. In addition, a research on the influence of personality on the usage by exploring a number of interaction types was conducted [25]. Frequency of available functions, which are posting photos of others, commenting on others’ photos, posting on others’ “wall” posts, sending private messages, poking, joining groups, and participating events were all included in the study [25]. The influence of personality on the time, frequency, and regrets on Facebook usage was also studied by [26]. Interaction types of commenting on others’ photos, posting on others’ wall posts were included. Both research made attempts to specify different communication means that are particular in Facebook using survey methods. Yet, self-reporting of usage behaviors still has its limitations in that recall problems of the respondents remain, and further, in that the data do not really show interaction between friends because interaction requires more than one party, while only one respondent reports her behavior in surveys.

Facebook users show less deceptive self-presentation online and tend to show who they *really* are [27, 28]. Therefore, user profiles uploaded on Facebook are considered more honest and trustful than in other SNSs [24, 29], making data on Facebook more appropriate for a data-driven research. Also, by analyzing behavioral data stored on Facebook, various interaction types can be taken into consideration altogether. However, few studies on Facebook actually collected activity log data in order to study interaction behaviors of Facebook users. In a study of developing a predicting model of tie strength between friends based on various information presented on Facebook, Gilbert and Karahalios incorporated various aspects of Facebook usage such as “days since last communication,” “distance between hometowns,” “inbox intimacy words,” and specific interaction types such as “participant-initiated wall posts,” “inbox messages exchanged,” “participant’s status updates,” “friend’s photo comments,” “appearances together in photo,” and “links exchanged by wall post” [30]. In a more recent research of estimating the relationship strength based on user attributes and interactions, wall posts and photo tagging activities were studied [22]. Even though the prior two research provided insights and interesting results, the most common and active interaction types on Facebook such as liking photos, commenting on photos, liking statuses were neglected in the paper.

Therefore, the second research question of current research is established in order to provide exploratory findings on the relation between diverse interaction behaviors and the quality of relationships, based on computer data logs of Facebook user interaction; other than interviews or survey questionnaires that show large discrepancies between self-reports and actual behaviors [3].

**Research Question 2.** How do communication behaviors in various interaction types—available on Facebook—correlate to the intimacy level of the user?

## Methods

The experiment included two procedures, survey and data crawling, and was conducted under the approval of the Seoul National University(SNU) Research Ethics Team (IRB No. 1305/001-022). A survey was conducted in order to measure the psychological closeness users feel towards each of their friend. Also, to measure similarities and interaction levels between people, users’ profiles and activity log data (between users and each of their friend) were crawled through Facebook API.

### Participant

Participants for the experiment were limited to Facebook users and were recruited through snowballing techniques from online channels. Recruiting Messages were posted in popular online websites and campus community sites with a brief explanation on the study and a link leading to the experiment website. A quote asking to share the post was also attached to the messages. Details on recruiting participants are in S1 Text.

### Experiment procedure

A web application using Facebook API was specifically built for the experiment. The application was built with Ruby on Rails and was designed to collect participants’ actual Facebook activity data (data crawling) and their responses (survey). On both of the recruiting message and the informed consent, the purpose of the study and where the collected data will be used were explicitly stated. For those who follow the link included in the recruiting flyer and sign in with their Facebook account, a pop-up screen requesting access to personal information and activity log data on Facebook appears. Data intended to be crawled were listed in detail on the pop-up. Survey was initiated only for those who grant permissions. Specific information allowed to be collected by participants is described in S2 Text.

The experiment website (Facebook web application) was open to public for 5 days and total number of 75 Facebook users participated in the study. Among the 75 participants, those who failed to identify either one of 3 fake friend and those who restricted access from all Facebook applications, and those who did not finish the entire survey were all excluded from the analysis. The final number of participants included in the study was 36. Additionally, 5 Facebook friendships were removed due to data collection issues (e.g. one of the 36th participant’s friends prevented access from Facebook applications). The final dataset for analysis contained 967(= 36x27-5) Facebook friendships.

#### Data crawling

Once a permission to collect data is granted by the participant, the web application we built accesses data of the participant’s Facebook user account via OAuth authentication provided by Facebook. OAuth is an authentication method that is commonly used in online applications, which enables applications to not only verify users but also access information on user profile and activity log data on Facebook through the account [31]. Specifically, the application accessed information on user profile and activity log data on Facebook through the account. Then, the participant was instructed to begin a survey on 30 of their randomly selected Facebook friends, including 3 fake friends to check data validity. A subset of 27 real friends was extracted randomly among participants’ Facebook friends who had interactions with the participant in 50 recent posts.

#### Survey

Then participants—who agreed to data collection—were asked to rate the intimacy level they feel towards each of the 27 randomly selected friends of theirs and 3 fake users: 3 fake users were randomly selected among a list of 10 fake users created in order to exclude unreliable participants from the study. All of the evaluated friends were listed with their Facebook account names and profile pictures so that participants could better identify their friends.

### Measures

*Similarity* indicates sharing personal attributes between a participant and a friend. It has been found that on average, users complete 59% of the user profile fields available to them [32]. Especially, only a small number of users listed their political views, religion, and marriage status, resulting in missing values of the shared values on them; thus, the three attributes were excluded in the study. In order to measure similarities in *Research Question 1*, data on Facebook user profiles of all 36 participants and subsets of their friends (27 for 31 participants, and 26 for 5 participants) were collected. In case of gender, location, hometown, work, high school, college, graduate school, concentration, when a participant and a friend share a certain attribute, it was coded as 1, and 0 otherwise. Age was regarded as the age difference between a participant and a friend. Groups, liked pages, and mutual friends were counted as numbers.

*Interaction* refers to activities that occur between users on Facebook. All activity types, i.e. likes, comments, tags, between 967 relationships are considered in the study; activities, such as pokes, that are only stored for a limited time frame are excluded in the study. Interaction behaviors that occurred on timeline were collected based on 300 recent posts. Distinct from interactions on timeline, message data (inbox/outbox) containing both synchronous chatting and asynchronous messaging are only collectable in message box threads based on the subjects of each chatting. Also, unlike posts uploaded on timeline, messaging only includes one specific activity type, i.e. exchanging private messages. Thus, 50 recent message boxes and individual messages within the thread of each of all 36 participants were crawled. Then, among the data, message exchanges only with the suggested friends of each participant were separately collected for analysis. Total amount of data collected and analyzed on each activity type were 10,464 comments, 16,171 likes, 569 tags, and 47,285 chats for all 967 relationships.

Interaction types are specified into the combination of post types and activity types, yet primarily based on the activity type. For analysis, while the activity of tags is specified into post types, likes and comments activity types incorporate all post types. The rationale for the classification is that tags vary in the form according to post types. For example, tags on photos are often done directly to the photo, identifying individuals by the tags, sharing a photo taken together or showing that they are/were physically together. On the other hand, tags on statuses are often used as sending direct message to certain people in a more public way or telling people that they are/were together. Moreover, tags on photos are presented on the photo while on statuses are only presented as texts.

The overall level of intimacy on each friend was measured by the ratings of 7 different items, which were adopted from the literature [33]; the items had alpha reliabilities of.93 in explaining psychological closeness. Identical subset of friends and strangers was suggested across the 7 items, which were all measured in a 7-point Likert scale with 1 for the least intimate, 7 for the most intimate, and 0 for “don’t know the person.” Specific questionnaires include “How close are you to your friend?”, “How much do you like your friend?”, “How often do you talk about personal things with your friend?”, “How important is your friend’s opinion to you?”, “How satisfied are you with your relationship with your friend?”, “How much do you enjoy spending time with your friend?”, and “How important is your relationship with your friend?”

## Results

Correlations between the survey data (intimacy) rated by participants and the data collected via the experiment website (similarity and interaction) are examined through multilevel analyses—random intercept models—since the range of rated intimacy scores varies according to each participant’s subjective assessment. Intimacy scores, evaluated by 31 participants towards each of their 27 friends and 5 participants towards 26 friends, are taken as the dependent variable for the study. In all 967 relationships, intimacy scores were measured as the mean score of 7 survey items. Initially, how similarity as an independent variable correlates to intimacy is explored. Then, in order to explore what interaction behaviors between Facebook users enhance the psychological closeness, how interaction as an independent variable correlates to intimacy is explored.

### Descriptive analysis

Descriptive analysis on the participants shows that there are twice more females (N = 24) than males (N = 12). Age distribution was highly skewed towards the 20s, averaged 24.4 (SD = 4.7), indicating that the result of current study is strongly limited to the Facebook users in 20s. Yet, biased features of the sample on Facebook users are not solely due to the sampling method in current research. Rather, they can be partly regarded as the demographic representing the Facebook community. The supposition is also supported by findings from a recent study, where Wells and Link (2014) discovered that the demographic patterns of Facebook users are more likely to be women, teens, whites, and adults with at least a high school diploma. Moreover, all participants in this study reported that they use Facebook at least once a day. Specifically, 75% were “always on” and 22.2% used Facebook more than 3 times a day. While 41.7% spent 10 to 30 minutes and another 41.7% spent 30 to 120 minutes a day on Facebook, only one participant spent less than 10 minutes and 13.9% spent more than 120 minutes. The basic information of participants demonstrates that people habitually open and check Facebook time after time, either to communicate with others or to kill time, rather than allocating a certain amount of time solely to Facebook, separate from other daily activities.

On Facebook, various communication methods are available for users. Overall, 4 activities (post_likes, comment_likes, post_comments, comment_tags, tags) can be performed on 4 different post types (link, video, photo, status) respectively, and a single messaging activity in chat. Link (N = 2,009, M = 2.08, SD = 6.7) and video (N = 27, M = 0.03, SD = 0.2) posts are seldom used as communication channels between participants and friends. In particular, tags activities on link and video types both never occurred among the participants’ relationships in current study. On the other hand, photos (N = 12,284, M = 12.7, SD = 24.9) and statuses (N = 12,884, M = 13.3, SD = 22.7) were actively used between friends. Likes (N = 16,171, M = 16.7, SD = 27.4) appeared to be the most popular way to communicate regardless of post types, followed by comments (N = 8,491, M = 8.8, SD = 20.9). Among many different possible communication channels on Facebook, likes and comments on photos and statuses were the most actively used interaction types on Facebook; likes (N = 7,806, M = 8.1, SD = 14.9) and comments (N = 3,247, M = 3.4, SD = 11.7) on photos, and likes (N = 6,352, M = 6.6, SD = 10) and comments (N = 4,680, M = 4.8, SD = 11.3) on statuses. Though messaging (N = 47,285) occurred most frequently, the mean and standard deviation (M = 48.9, SD = 320.8) of participants’ messaging activities indicate that some participants actively used messages while some barely used messages.

### Similarity on attributes that predict intimacy

Similarity is measured by shared attributes between a participant and a friend. Attributes that compose similarity are taken into consideration based on Facebook user profile information of each user. Since how much information the user puts up on the profile page fully depends on the user’s decision, there were missing values across attributes. Assuming that there is no similarity that a user would recognize toward his friend when information on certain attribute of the user—such as workplace—is not exhibited on the profile page of a friend, missing values (NA) were treated as 0.

The result of a multilevel analysis on similarity (independent variable) and intimacy (dependent variable) is summarized in Table 1. Unlike findings from prior studies, results based on female Facebook users in their 20s, no significant correlations were found between attribute similarities and intimacy. Only having attended the same graduate school and having mutual friends showed statistically significant, yet low, correlations to intimacy.

**Table 1:** A multilevel analysis result of similarity.

Similar User Attributes	*β*	*σ*	*p*	*Sig*.^a^
(Intercept)	3.333	0.169	<0.001	***
co_gender	0.152	0.130	0.240	
age_difference_by_year	−0.002	0.018	0.923	
co_location	0.051	0.154	0.742	
co_hometown	−0.103	0.183	0.574	
co_work	0.309	0.341	0.366	
co_highschool	0.016	0.182	0.931	
co_college	−0.045	0.146	0.760	
co_gradschool	0.843	0.375	0.024	*
co_concentration	−0.103	0.218	0.635	
co_groups	0.055	0.058	0.342	
co_liked_pages	0.009	0.008	0.255	
mutual_friends	0.004	0.002	0.027	*

^a^* p<.05,

*** p<.001

### Interaction types that predict intimacy

After descriptively looking into the general picture of users’ interaction behaviors on Facebook, a multiple linear regression analysis was conducted to identify interaction types that enhance intimacy between participants and their friends (see Table 2). Interaction types are measured as frequencies by dividing each activity type, separated by cases where a participant is the receiver (from_friends) and where the participant is the sender (to_friends).

**Table 2:** Multilevel analyses results of interactions on timeline and messenger.

Interaction Types	*β*	*σ*	*p*	*Sig*.^a^
(Intercept)	3.587	0.109	<0.001	***
likes_from_friends	−0.004	0.007	0.529	
likes_to_friends	0.001	0.005	0.752	
comments_from_friends	0.015	0.007	0.026	*
comments_to_friends	0.002	0.005	0.732	
photo_tags_from_friends	0.117	0.089	0.191	
photo_tags_to_friends	−0.012	0.041	0.772	
status_tags_from_friends	0.488	0.306	0.111	
status_tags_to_friends	0.904	0.294	0.002	**
(Intercept)	3.711	0.102	<0.001	***
chat_from_friends	0.003	0.001	0.013	*
chat_to_friends	−0.002	0.001	0.060	.

^a^. p<.1,

* p<.05,

** p<.01,

*** p<.001

Likes had no statistically significant correlation with intimacy, regardless of the direction; both likes_from_friends and likes_to_friends. The most significant interaction type turned out to be comments_from_friends, with a high beta coefficient, while comments_to_friends did not show any statistic significance on intimacy and had a low beta coefficient. The result indicated that participants felt more intimate to friends who made comments frequently to the participant. Moreover, comments made from the participant to a friend did not predict intimacy the participant felt towards the friend. This could be put in another way: though a user tend to feel more intimate to friends who make comments to the user, the user does not necessarily make more comments to friends that the user feels more intimate to. Status_tags_to_friends was positively correlated to intimacy with a relatively high beta coefficient. That is, participants tend to directly tag their friends, to whom they feel more intimate.

Chat activity on the messenger shows an interesting finding. Chat_from_friends had a significant correlation to intimacy, with a positive beta coefficient. The significance indicates that the more a friend sends chat messages to a participant, the more intimate the participant would feel towards the friend; similar to the case of comments_from_friends. In contrast, chat_to_friends showed a negative correlation to intimacy even though the significance was low. Thus, interpretations on the interaction type of sending chat messages from and to friends require further elaboration.

## Discussion

Main findings from current research are related to managing relationships that are formed or maintained online in the context of Facebook. While strong inferences cannot be drawn due to skewness of the sample analyzed in this paper, results on the interaction data give opportunities to explore dynamic features of communication behaviors on Facebook.

In Facebook networks, most of the similarities on user profile showed no significant level of correlations with intimacy. Only two attributes, having gone to the same graduate school and having mutual friends significantly promoted the psychological closeness; yet low. Considering that some studies show that education, occupation, and social class tend to have greater effects on less intimate relationships [2, 5], it is possible to presume that personal networks on Facebook are not so intimate. One possible interpretation on the result is that in online contexts such as Facebook, people often find cues or get to know about other people through their profile pages. Unlike in contexts such as offline or videoconferencing, users cannot literally *see* and *meet* in person on Facebook. Thus, they are unable to confirm or realize the similarity between each other. As a result, similarities in personal attributes may become relatively less substantial. Moreover, users often find cues or get to know about others through their profile pages. Yet, content on profile pages are registered by the friends themselves, and therefore, there is a high possibility that users would not give much credit or value to the information exhibited on the page, i.e. Facebook profile.

Results on comments suggest that though a user tend to feel more intimate to friends who write comments to the user, the user does not necessarily make more comments to friends that the user feel more intimate to. A careful speculation on the behavior is that people tend to use other personal channels that are more exclusive in order to contact close *friends*, such as short messaging service(sms), WhatsApp, and Viber. Likes activity is the most frequently and commonly used channel in Facebook, but it showed no significant correlation to intimacy. Since likes require the least effort to engage, participants may not necessarily relate the activity to the psychological closeness they feel towards their friends. Moreover, likes indicate agreement on attitudes or opinions to a certain post rather than proving “how much I *like* you” to a friend who uploaded the post.

Interesting results were obtained on photo tags. Intuitively, if people are tagged together in a photo, or if either one of the person tags the other, the relationship seems to be close. However results in the study do not support the supposition. Despite the necessity of further inquiry on the issue, it may be due to web application services provided on Facebook that publish photos with users’ Facebook friends tagged in it; for example, a calendar photo with all birthdays of Facebook friends being tagged in the calendar. Findings from chat in current research could be understood as Facebook being primarily used as a communication service between acquaintances, with a specific purpose of “keeping the relationship alive” or “checking” with friends who are neither close nor a stranger. Those in confiding relations may not care or need to know about friends’ news via Facebook, because they already know. Moreover, there are many other private or instant communication services available for those in close relationships other than Facebook messenger, such as instant messaging, SMS, phone calls, meeting offline etc. Even among online channels such as iMessenger, sms, Skype, LINE, and WhatsApp, Facebook messenger is possibly the least effective among those: it does not appear to be the primary channel for one-to-one conversations.

## Conclusion

Taking the perspective of [1], looking into relationship as a process, keeping relations could be studied in terms of “seeking continued contact,” i.e. interacting. In Facebook, there are various ways to interact in order to keep in touch with others. Quality of a relationship that is maintained online is highly influenced by how the other makes contact to an individual. In other words, in an online context, selecting and employing the *right* communication method may be the key in relationship management. Accordingly, an investigation on correlations of diverse interaction types and intimacy in Facebook was conducted, and as a result, certain interaction types appeared to have more significance in relations to intimacy.

A more fundamental issue could be addressed from the results of current research. Considering that most of the interaction types appeared to have no significant correlation with intimacy, Facebook could be a space where intimacy is not the key in the process of developing and maintaining relationships. Rather, expressing and presenting oneself to others may be in the center of interest among users [34, 35]. In an ethnographic study of boyd (2007), Facebook is described as a place where users feel disconnected to their friends and where friendships are often established reluctantly with acquaintances, only because of a social norm that refusing friend request is considered impolite [28]. Also, it has been found that many Facebook users feel annoyed and hoped their friends to keep away from too much personal emotional exposure on Facebook, indicating less emotional closeness to friends [36]. Though Facebook may have started its service as a small community, where the network represented a rather closed and intimate social circle, as the service became open to public, the number of users and networks formed in Facebook has grown enormously in size, making Facebook a space full of narcissism. Thus, it is likely that people who seek to have private and intimate conversations and who appreciate quality of relationships adopt other less popular online channels to keep in touch.

Also, findings from current study suggest further elaboration on relationship management by comparing groups of people based on the quality of relationships. Especially, results from the first research question on the non-significant correlation between attribute similarities and intimacy, as well as results from the second research question on the interaction type of “chat,” enable a speculation on the relationship-based difference in interaction behaviors. According to established literature, also the influence of similarities in personal attributes on intimacy differs across a wide range of relationships, such as superficial relations, close friendships, confiding relations, and kinship [3, 37]. Likewise, categorizing relationships based on intimacy scores towards friends and investigating communication behaviors according to the distinct level of relations could facilitate a more thorough understanding on computer-mediated communication.

Further, there are some issues to be re-considered and improved in current research. First issue concerns random selection of participants’ friends for survey. Facebook friends of each participant were randomly selected based on two reasons. First of all, selecting friends based on the similarity of personal attributes listed in Facebook user profile was impracticable, since no data on participants or their friends were available beforehand. Also, deciding friends according to the interaction behavior between the participant and the friend on Facebook was fundamentally absurd, because no theoretical expectations or sound evidence were established on the relationship between intimacy and various interaction types used as communication channels on Facebook. Secondly, the sample size itself is not representative of the Facebook user population. The sample size of 36 had highly skewed age distribution, focused on the 20s. Though the result cannot be applied across ages without further inquiry, more than 70,000 interactions among 967 Facebook friendships were analyzed. In addition, unlike earlier literature on Facebook usages and interaction behaviors that were focused on investigating the frequency of interaction on the usage, this study looked into interaction behaviors more in detail by distinguishing the sender (“from”) and receiver (“to”) of every interaction behaviors between 967 friendships, and included the factor in analyses. Current paper provides insights and opportunities to understand relationships from a different perspective that is more relevant to the computer-mediated context. Nevertheless, a larger and a more representative sample of the population could have brought more interesting and insightful findings or data-driven patterns to the work. Hence, it is necessary to carry out follow-up studies with a more representative sample on Facebook, so that more profound inferences and understandings could be drawn from analyses.

Another issue concerning the sample is numerous missing values of user profile information on Facebook. A number of users have a number of missing information in their profiles, making the factor uncontrollable by researchers. Yet, such matter is also inherent in data-driven analyses. While data-driven research has its strength in that it gives an opportunity to gain insights that are not easily predictable by depending on the literature alone, it has its weakness in that the analysis fully depends on the collected data. Recruiting Facebook users who registered all sections of profile attributes would have provided a better understanding on the relationship between user similarities and intimacy. Nonetheless, in a different perspective, such massive missing information can be understood as a more selective self-presentation in online space. Selective self-presentation itself manifests the nature of Facebook [35] and it could be the case in any other online settings as well. Selective or strategic self-presentation is often used in order to display an ideal self, to create positive impressions, to alleviate negative impressions, or to induce empathy and compliance [34, 35, 38]. While prior studies on self-presentation focused on the uploaded information online, *not* uploading certain information, e.g. keeping one’s age as a secret is also a strategica way to present oneself; not letting others know about herself to some extent.

Additionally, causal relationship on the intimacy and interaction can be questionable. This is an inevitable limitation on cross-sectional studies. Especially, considering the social influence process, which demonstrates that people who associate tend to become similar, interaction behaviors may be the cause of the psychological closeness, not the result. Such suspicion is a more fundamental question on the whether it is the psychological process or the social influence process that explains people’s relationship better [22]. The question on the causal relationship should be further investigated in future studies based on a longitudinal data.

Despite the limitations, current research makes its contribution in certain aspects. This paper explores whether a tendency prevalent in face-to-face contexts, i.e. people feeling more close to the similar ones, is also applicable to the computer-mediated context. Findings show that unlike in offline contexts where people meet in person, it is not the personal attributes (sex, age, race etc.), but the way others talk and keep in touch (interaction behaviors) that becomes visible and prominent. Hence, the paper suggests to examine the quality of relationships developed and maintained in the online context based on interaction behaviors. In doing so, current study diminished the bias that may occur from recall by collecting activity log data rather than relying on self-reported data. Moreover, current work investigates distinct interaction methods available in the online space, such as tags and likes, and further, takes the direction of interaction into consideration as well, e.g. “did the user receive likes from a friend or did he send likes to the friend?” Based on findings and insights from current research, by collecting data on a larger number of users for a longer period across different online channels, deeper knowledge in the way people manage relationships and how the quality of relationships changes over time could be provided in future work.

## Supporting information

S1 Text Participants were initially recruited via an online campus website of Seoul National University, Facebook, and Twitter. It was explicitly stated on the recruitment flyer and the informed consent that the collected data on profile information, survey questionnaire, and interaction behavior will be used in order to explore the relationships between similarity-intimacy, and intimacy-behavior.
(PDF)

S2 Text A message asking for permission to access data included lists of information that would be crawled once the participant grants the access. Specifically, public profile, friend list, email address, custom friends lists, messages, News Feed, relationships, birthday, work history, status updates, education history, groups, hometown, current city, photos, religious and political views, videos, personal description, likes and your friends’ relationships, birthdays, birthdays, work histories, status updates, education histories, groups, hometowns, current cities, photos, religious and political views, videos, personal descriptions and likes were listed.
(PDF)
