# Supplementary material for: With whom do you feel most intimate?: Exploring the quality of Facebook friendships in relation to similarities and interaction behaviors
Source: PLoS One. 2017 Apr 28;12(4):e0176319. doi: 10.1371/journal.pone.0176319 (PMC5409138; doi:10.1371/journal.pone.0176319)
Supplement: S1 Text — It was explicitly stated on the recruitment flyer and the informed consent that the collected data on profile information, survey questionnaire, and interaction behavior will be used in order to explore the relationships between similarity-intimacy, and intimacy-behavior. (PDF) [file pone.0176319.s001.pdf]

Supporting Information

S1. Details on Recruitment

Participants were initially recruited via an online campus website of Seoul National University, Facebook, and Twitter. It was explicitly stated on the recruitment flyer and the informed consent that the collected data on profile information, survey questionnaire, and interaction behavior will be used in order to explore the relationships between similarity-intimacy, and intimacy-behavior.
