# Supplementary material for: With whom do you feel most intimate?: Exploring the quality of Facebook friendships in relation to similarities and interaction behaviors
Source: PLoS One. 2017 Apr 28;12(4):e0176319. doi: 10.1371/journal.pone.0176319 (PMC5409138; doi:10.1371/journal.pone.0176319)
Supplement: S2 Text — Specifically, public profile, friend list, email address, custom friends lists, messages, News Feed, relationships, birthday, work history, status updates, education history, groups, hometown, current city, photos, religious and political views, videos, personal description, likes and your friends’ relationships, birthdays, birthdays, work histories, status updates, education histories, groups, hometowns, current cities, photos, religious and political views, videos, personal descriptions and likes were listed. (PDF) [file pone.0176319.s002.pdf]

Supporting Information

S2. Details on Access to Data

A message asking for permission to access data included lists of information that would be crawled once the participant grants the access. Specifically, public profile, friend list, email address, custom friends lists, messages, News Feed, relationships, birthday, work history, status updates, education history, groups, hometown, current city, photos, religious and political views, videos, personal description, likes and your friends' relationships, birthdays, birthdays, work histories, status updates, education histories, groups, hometowns, current cities, photos, religious and political views, videos, personal descriptions and likes were listed.
